# The Associations of Fruit and Vegetable Intake with Lung Cancer Risk in Participants with Different Smoking Status: A Meta-Analysis of Prospective Cohort Studies

**DOI:** 10.3390/nu11081791

**Published:** 2019-08-02

**Authors:** Chong Wang, Ting Yang, Xiao-fei Guo, Duo Li

**Affiliations:** 1 Institute of Nutrition and Health, Qingdao University, Qingdao 266071, China; 2 Department of Food Science and Nutrition, Zhejiang University, Hangzhou 310058, China

**Keywords:** fruit, vegetable, lung cancer, prospective study, meta-analysis

## Abstract

The results of epidemiological studies on the relationship between fruit and vegetable intake and lung cancer risk were inconsistent among participants with different smoking status. The purpose of this study was to investigate these relationships in participants with different smoking status with prospective cohort studies. A systematic literature retrieval was conducted using PubMed and Scopus databases up to June 2019. The summary relative risks (RRs) and the corresponding 95% confidence intervals (CIs) were calculated by random-effects model. The nonlinear dose-response analysis was carried out with restricted cubic spline regression model. Publication bias was estimated using Begg’s test. Nine independent prospective studies were included for data synthesis. Dietary consumption of fruit was negatively correlated with lung cancer risk among current smokers and former smokers, and the summery RRs were 0.86 (95% CI: 0.78, 0.94) and 0.91 (95% CI: 0.84, 0.99), respectively. Consumption of vegetable was significantly associated with reduced risk of lung cancer for current smokers (summary RR = 87%; 95% CI: 0.78, 0.94), but not for former smokers and never for smokers. Dose-response analysis suggested that risk of lung cancer was reduced by 5% (95% CI: 0.93, 0.97) in current smokers, and reduced by 4% (95% CI: 0.93, 0.98) in former smokers with an increase of 100 grams of fruit intake per day, respectively. Besides, dose-response analysis indicated a 3% reduction in lung cancer risk in current smokers for 100 gram per day increase of vegetable intake (95% CI: 0.96, 1.00). The findings of this study provide strong evidence that higher fruit consumption is negatively associated with the risk of lung cancer among current smokers and former smokers, while vegetable intake is significantly correlated with reducing the risk of lung cancer in current smokers. These findings might have considerable public health significance for the prevention of lung cancer through dietary interventions.

## 1. Introduction

The data from the International Agency for Research on Cancer indicate that lung cancer is the primary reason of cancer incidence and mortality, accounting for an estimated 2.1 million new cases of lung cancer and 1.8 million deaths worldwide in 2018 [1]. In terms of gender, lung cancer is the most common diagnosed cancer and the leading cause of cancer death in men. Meanwhile, lung cancer is the second most common cancer in women and a secondary cause of cancer mortality. Cigarette smoking is considered as the main cause of lung cancer. The lung cancer incidence is higher in men, and may be caused by the higher consumption of cigarettes compared to women [2]. So far, smoking is still the principal reason of lung cancer. Smoking cessation is the most direct and effective way to prevent lung cancer [3]. However, because smoking is addictive, it is difficult for long-term smokers to quit smoking. Therefore, there is an urgent need for an effective and safe way to reduce the risk of lung cancer among smokers. There is now a growing focus on diet and nutritional interventions for cancer prevention. Synchronously, changing lifestyles and dietary habits have shown to be a practical way to reduce the risk of lung cancer [4]. Of these, vegetable and fruit have been paid increasing attention because of their rich essential nutrients and antioxidant substances.

Meta-analysis methodology was used to systematically analyze the associations of fruit, vegetable or fruit and vegetable combined intake with lung cancer risk [5,6,7], however no meta-analyses focused on the associations between fruit and vegetable intake and lung cancer risk in participants with different smoking status. The results from the European Prospective Investigation into cancer and nutrition have suggested an inverse correlation between fruit and vegetable consumption and lung cancer risk in current smokers [8]. Whereas in the Nurses’ Health Study and Health Professionals’ follow-up study, vegetable and fruit showed to have a protective effect on never-smoking men and women [9]. Additionally, in the JPHC cohort study, fruit and vegetable were not associated with smoking status (never or ever smokers) [10]. Therefore, it is urgent and necessary to comprehensively evaluate these associations. Due to the diverse chemical components of fruit and vegetable, such as dietary fiber, inorganic salts, phytochemicals and vitamins, they would have different potentials for the initiation and development of lung cancer. Therefore, we performed a comprehensive meta-analysis to elucidate the correlations between vegetable and fruit intake and lung cancer risk in participants with different smoking status.

## 2. Materials and Methods 

### 2.1. Study Selection

We followed the standard for conducting and reporting meta-analysis of observational studies [11]. PubMed and Scopus databases were used to conduct a systematic retrieval of the literature before June 2019. Fruit or vegetable was paired with cancer, tumor, carcinoma, neoplasm or lung cancer as search term. The original studies were limited to English-language publications. Additional manual searches were performed using reference lists from original research papers, previous meta-analyses and reviews.

### 2.2. Eligible Criteria

The inclusion criteria were as follows: (1) prospective studies, which included nested case-control, case-cohort and prospective cohort studies; (2) the exposure of interest, which were dietary intakes of vegetable or fruit; (3) the original studies, which provided the relative risks (RR) with the 95% confidence intervals (CIs) in participants with different smoking status; and (4) the outcome of interest, which was lung cancer. When multiple publications of the same study were published, we used the publication with the maximum quantity of cases, the most applicable information and the most recent publications.

### 2.3. Data Extraction and Quality Assessment

For identified studies, data extraction was performed independently by two researchers (C.W. and T.Y.), and any conflict was settled via discussion reaching a consensus. Extracted data, comprised first author, nation/country where the study was conducted, published year, age, gender, sample size, number of cases, amount of fruit or vegetable intake, exposure measurement, measurement of outcomes, RRs and 95% CIs and adjusted confounders. In each study, the multivariate-adjusted RRs with the 95% CIs were extracted. In the studies included, the consumption of fruit and vegetable was measured in different ways and in various units, for instance; gram per day and serving per week. We normalized all data into gram per day. First, we translated serving from per week to per day. Then, using 106 grams as standard portion size the unit of serving per day was transformed into gram per day by multiplying 106 [12]. If two investigators disagree on eligibility data, they would decide by the third reviewer.

Newcastle-Ottawa Scale was used for quality assessment. [13]. The scoring system summarized nine aspects of each study. The highest rating is 9 stars, and 0–3, 4–6, 7–9 was categorized as low, medium and high-quality, respectively.

### 2.4. Statistical Analysis

Using the random-effects model, we pooled the RRs as weighted by reciprocal of the variance. The highest versus lowest categories of study-specific RR were used to assess this correlation. The highest level of fruit and vegetable intake was extracted. If the maximum quantile was unlimited, the dose was defined as 1.2 times of the maximum boundary. The relationship between fruit and vegetable with lung cancer risk was evaluated by using a two-stage random-effects dose-response analysis. To evaluate the potential curvilinear (non-linearity) correlations between fruit and vegetable and lung cancer risk, we simulated the dose using a restricted cubic spline model with three knots on the distribution percentages (25%, 50% and 75%, respectively) [14]. By testing the null hypothesis that the regression coefficient of the second spline was equal to zero, the P value of the potential curve was calculated [15]. In the case of a significant linear trend (*p*-value for curvilinear > 0.05), a linear dose-response meta-analysis of trend estimation was performed using the generalized least squares regression method proposed by Greenland and Longnecker (1992) [16] and Orsini et al. (2006) [17] to assess the relationship between increment of fruit and vegetable intake and lung cancer risk.

Heterogeneity among studies was estimated using I^2^ statistic. The I^2^ values of 25%, 50% and 75% as cut-off points indicate low, moderate and high levels of heterogeneity, respectively [18]. To explore whether the results were strongly influenced by any specific study, sensitivity analyses were performed—excluding one study at a time. Publication bias was conducted through Begg’s test, and was a significant representative test of publication bias. Statistical analysis was performed with STATA 11.0 for Windows (Stata CORP, College Station, TX). The *p*-value was two-tailed, with a significant level of 0.05.

## 3. Results

### 3.1. Literature Search and Study Characteristics

**Figure 1:**
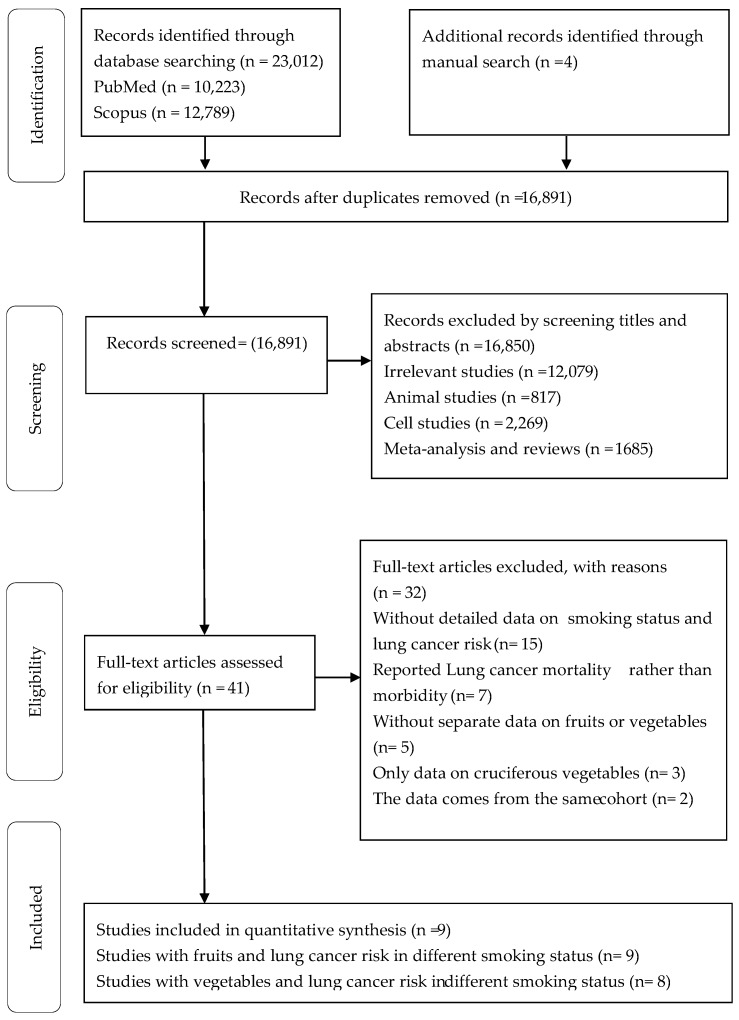
PRISMA Flow Diagram of the study selection procedure showing the number of eligible trials included in the present study.

The flow diagram of the literature retrieval is presented in Figure 1. We identified 10,223 citations from PubMed, 12,789 from Scopus, and 4 from manual search. Of these, there were 16,891 articles left after eliminating duplicates. After reviewing the title and abstract, we excluded articles irrelevant to animal experiments, cell experiments, meta-analysis and systematic review, and retrieved the full text of 41 articles to evaluate whether they met the inclusion criteria. After full-text examination, 32 articles were excluded because they did not meet the inclusion criteria (15 did not provide data on smoking, seven reported lung cancer mortality rather than morbidity, five did not have data on individual vegetable or fruit, three reported only the relationship between cruciferous vegetable and lung cancer risk, and two data comes from the same cohort). Finally, we identified nine articles that met the inclusion criteria [8,9,10,19,20,21,22,23,24]. 

The characteristics of included studies are listed in Table 1. The article of Feskanich et al. [9] included two cohort studies, namely the Nurses’ Health Study and the Health Professionals’ Follow-up Study. The research of Liu et al. [10] was separated into the Japan Public Health Center-based prospective study cohort I and cohort II. Two articles [22,23] were divided into male and female for analysis, respectively. Additionally, one study included eight cohorts and another study included four cohorts. Overall, a total of 12 independent cohort studies from nine articles for data analysis. Of the nine articles, four were conducted in USA, one in Europe, two in Japan, and one study is a pooled analysis including eight cohorts [19]. The duration of follow-up ranged from 4 to 12.9 years. According to the Newcastle-Ottawa Scale criteria, four articles were rated as medium quality and five were rated as high quality (Table 2).

### 3.2. Fruit and Lung Cancer Risk in Subjects with Different Smoking Status

**Figure 2:**
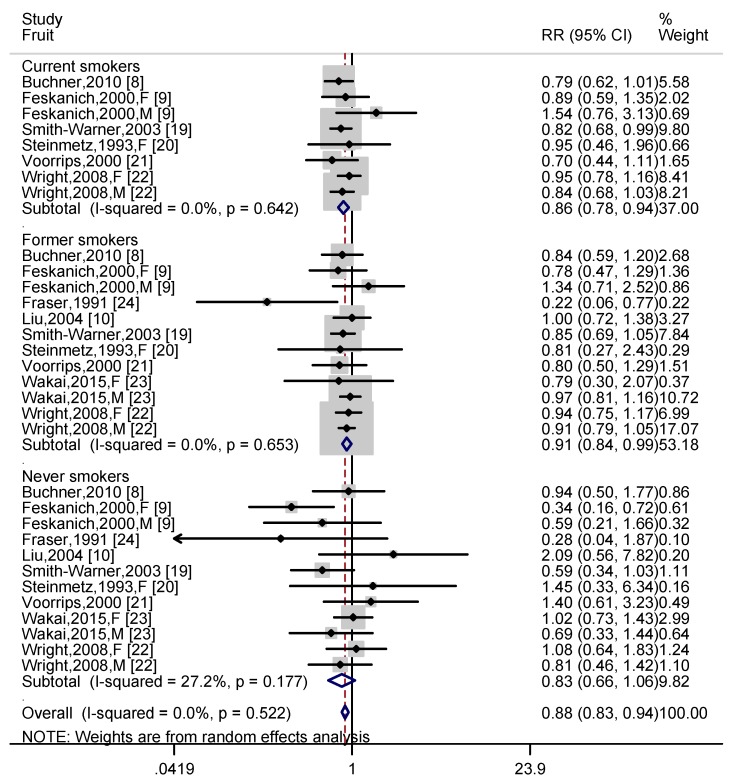
Differences in fruit composition between current smokers, former smokers and never smokers. The pooled effect was calculated using a random-effects model. The diamonds denote summary risk estimate, and horizontal lines represent 95% CI. Abbreviations: F—female; M—male; RR—relative risk.

Data on fruit intake and lung cancer risk in different smokers were obtained from nine articles [8,9,10,19,20,21,22,23,24]. Six independent prospective cohort studies [8,9,19,20,21,22] reported the relationship between fruit intake and lung cancer risk among current smoker. A higher intake of fruit was associated with 14% (RR = 0.86, 95% CI: 0.78, 0.94; I^2^ = 0.0%, *p* heterogeneity = 0.642) reduction of lung cancer risk. For former smokers, nine independent cohort studies [8,9,10,17,18,19,20,21,22] provided available data with reference to fruit. A higher intake of fruit was negatively correlated with the risk of lung cancer risk (RR = 0.91, 95% CI: 0.84, 0.99; I^2^ = 0.0%, *p* heterogeneity = 0.653). Nine independent studies [8,9,10,19,20,21,22,23,24] investigated the relationship between fruit intake and lung cancer risk in never smokers, and a higher fruit consumption showed a borderline significant association (RR = 0.83, 95% CI: 0.66, 1.06; I^2^ = 27.2%, *p* for heterogeneity = 0.177). Besides, the pooled effect of fruit on all smoking status subjects showed a significant reduction in lung cancer risk (RR = 0.88, 95% CI: 0.83, 0.94; I^2^ = 0.0%, *p* for heterogeneity = 0.522) (Figure 2). In addition, the risk of lung cancer among different smokers was stratified by region according to fruit intake. Stratified analysis showed that fruit intake significantly reduced the risk of lung cancer in current smoking subjects from Europe (RR = 0.77, 95% CI: 0.62, 0.96), but not for Americas (RR = 0.91, 95% CI: 0.80, 1.04). Among former smokers and never-smokers, fruit consumption had no statistically significant effect on lung cancer risk in European, American, and Asian participants (Figures S1–S3).

**Figure 3:**
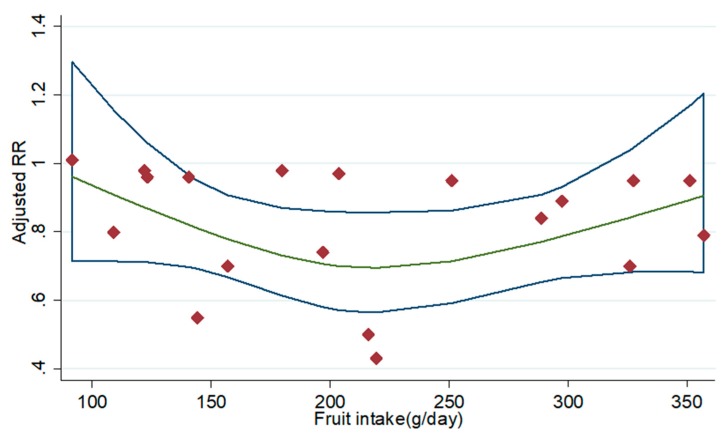
Dose-response analysis for the curvilinear association between intakes of fruit in current smokers and lung cancer risk. Abbreviations: RR—relative risk.

**Figure 4:**
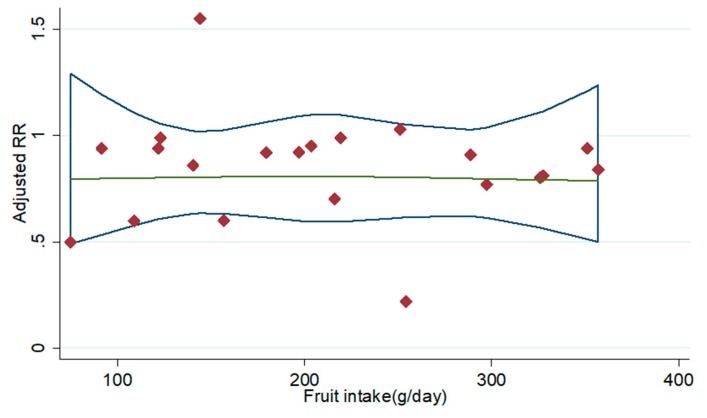
Dose-response analysis for the curvilinear association between intakes of fruit in former smokers and lung cancer risk. Abbreviations: RR—relative risk.

Four prospective cohort studies of current smokers [8,20,21,22] and five prospective cohort studies of former smokers [8,20,21,22,24] met the requirements of dose-response analysis, and non-significant curvilinear correlation was observed between fruit intake in current smokers (*p* for non-linearity = 0.395) and former smokers (*p* for non-linearity = 0.571) and lung cancer risk (Figure 3 and Figure 4). Whereas linear dose-response analysis suggested that an increase of 100 grams of fruit intake per day was associated with 5% reduction in lung cancer risk in current smokers (95% CI: 0.93, 0.97; *p* for trend <0.001) and 5% reduction in former smokers (95% CI: 0.97, 0.99; *p* for trend = 0.001).

### 3.3. Vegetable and Lung Cancer Risk in Subjects with Different Smoking Status

**Figure 5:**
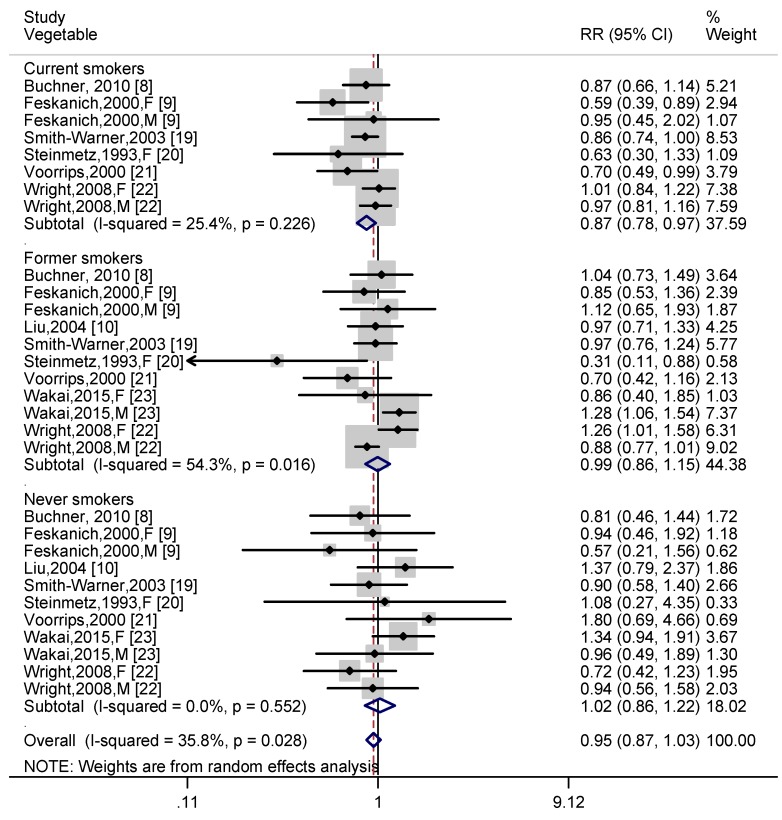
Differences in vegetable composition between current smokers, former smokers and never smokers. The pooled effect was calculated using a random-effects model. The diamonds denote summary risk estimate, and horizontal lines represent 95% CI. Abbreviations: F—female; M—male; RR—relative risk.

Data on vegetable intake and lung cancer risk in different smokers were obtained from eight articles [8,9,10,19,20,21,22,23]. Six independent prospective cohort studies [8,9,19,20,21,22] reported the association of vegetable intake with lung cancer risk in current smokers. For former smokers and never smokers, nine independent cohort studies [8,9,10,19,20,21,22,23] provided available data with respect to vegetables. As shown in Figure 5, a significant negative correlation was observed (RR = 0.87, 95% CI: 0.78, 0.97; I^2^ = 25.4%, *p* for heterogeneity = 0.226) in current smokers, but not for former smokers (RR = 0.99, 95% CI: 0.86, 1.15; I^2^ = 54.3%, *p* for heterogeneity = 0.016) and never smokers (RR = 1.02, 95% CI: 0.86, 1.22; I^2^ = 0.0%, *p* for heterogeneity = 0.552), and the pooled effect of vegetables on all smoking status subjects showed no statistically significant in lung cancer risk (RR = 0.95, 95% CI: 0.88, 1.03; I^2^ = 30.9%, *p* for heterogeneity = 0.042). In addition, the risk of lung cancer among different smokers was stratified by region according to vegetable intake. Stratified analysis showed that vegetable intake was associated with a reduction (RR = 0.80, 95% CI: 0.65, 0.99) in lung cancer risk among European participants, but not statistically significant (RR = 0.89, 95% CI: 0.74, 1.07) among subjects in the Americas. The effect of vegetable intake on lung cancer risk in European, American, and Asian participants were not statistically significant in former smokers or never smokers (Figures S4–S6).

**Figure 6:**
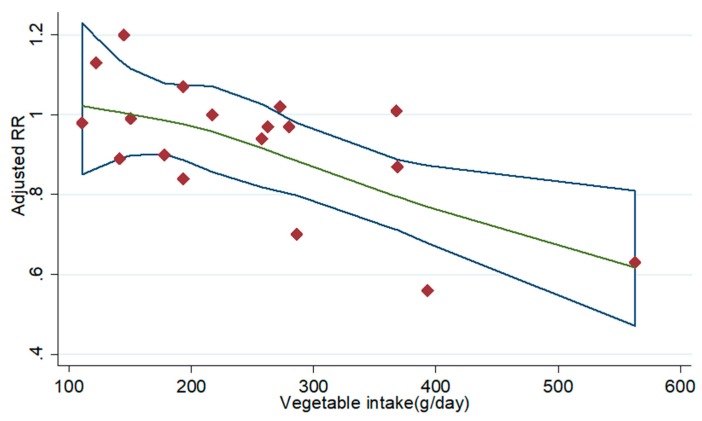
Dose-response analysis for the curvilinear association between intakes of vegetable in current smokers and lung cancer risk. Abbreviations: RR—relative risk.

Four prospective cohort studies met the requirements of dose-response analysis [8,20,21,22], observing a non-significant curvilinear relationship between vegetable intake in current smokers and lung cancer risk by using a restricted cubic splines models (*p* for non-linearity = 0.698). However, linear dose-response analysis suggested that an increase of 100 grams of vegetable intake per day was associated with a 3% lower risk of lung cancer risk in current smokers (95% CI: 0.96, 1.00; *p* for trend = 0.057) (Figure 6).

### 3.4. Sensitivity Analysis and Publication Bias

The results of the sensitivity analysis are presented in the supplementary material (Figures S7–S12), indicating that the pooled analyses were stable. Through Begg’s test, no publication bias was found with respect to fruit consumption in current smokers (*p* = 0.711), former smokers (*p* = 0.150) and never smokers (*p* = 0.837). No publication bias was found with reference to vegetable consumption in current smokers (*p* = 0.386), former smokers (*p* = 0.161) and never smokers (*p* = 1.000). 

## 4. Discussion

This meta-analysis found that fruit intake was associated with a 14% lower lung cancer risk in current smokers and a 9% lower lung cancer risk in former smokers, and the result in never smokers (RR = 0.83, 95% CI: 0.66, 1.06) were non-statistically significant. However, there was a significant negative relationship between vegetable intake and lung cancer risk only in current smokers (RR = 0.87, 95% CI: 0.78, 0.97), which was not observed in former smokers (RR = 0.99, 95% CI: 0.86, 1.15) and never-smokers (RR = 1.02, 95% CI: 0.86, 1.22). An increase of 100 grams of fruit or vegetable intake per day was associated with a 5% and 3% reduction in risk of lung cancer in current smokers, respectively.

Vegetable and fruit are essential food in daily life, because they contain various nutrients such as vitamin, mineral, phytochemical and dietary fiber, which might play an important role in the prevention of lung cancer. Our study found that higher intakes of vegetable and fruit were correlated with reduced lung cancer risk in current smokers. This may be due to the large amounts of free radicals in cigarettes, which cause cell and DNA damage, thus increasing the level of oxidative stress, and DNA oxidative damage is the central part of lung cancer [25]. At the same time, smoking can also cause lung inflammation and thus promote the development of lung cancer. On the contrary, higher intakes of fruit and vegetable, which contain various kinds of antioxidant active substances, such as vitamin E, vitamin C, beta-carotene and B vitamins, have shown antioxidant activates and repair DNA oxidative damage caused by smoking [26,27,28,29,30,31]. Isothiocyanates, indoles, flavonoids in vegetable and fruit and other phytochemicals could also regulate anti-tumor pathways through different mechanisms, inhibit tumor cell proliferation and induce tumor cell apoptosis, thereby reducing the risk of lung cancer [32,33]. Some ingredients in vegetable and fruit also have the function of regulating inflammatory reaction and reducing serum c-reactive protein and interleukin-6, so as to achieve the purpose of preventing lung cancer, such as vitamin A, vitamin E, polyphenols, organic sulfides, plant sterols and dietary fiber [32,34,35,36,37]. Certain types of vegetable, such as cruciferous vegetable and garlic vegetable, are rich in sulfide, which can reduce the risk of lung cancer [38]. 

The present study has several advantages. Primarily, to our knowledge, the current study is the first meta-analysis of the effect of vegetable and fruit as exposure factors on lung cancer risk in participants with different smoking status. Secondly, large sample size and strong ability of statistical results could more accurately estimate the relationship between the intake of vegetables or fruits and the risk of lung cancer in smokers of different status, and the summary estimates of the present study would be more credible. Thirdly, the sensitivity analysis indicated that the pooled estimates were not varied substantially after the deletion of any one study, indicating the stability of the pooled estimates. Meanwhile the results of Begg’s test indicate that there was no obvious publication bias, which also manifests the stability of the results. In addition, the dose-response analysis also provided a large amount of evidence that the intake of fruit and vegetable was negatively associated with the risk of lung cancer in a dose-dependent manner.

Contemporaneously, this study also has several drawbacks. The studies included in this meta-analysis were from the United States, Japan, the Netherlands and several countries in Europe, and were published from 1991 to 2015. The categories of vegetable and fruit defined in different regions, different populations and different periods may be various, so the results might not be comparable. Stratified analysis was performed, indicating that regions may be the source of heterogeneity. In addition, none of the studies included in this meta-analysis had a clear definition of smoking, including whether the types of cigarettes included traditional pipe and cigar. Because traditional pipe, cigar and cigarette smoking would lead to different cancer risks, and the former would lead to high risk of head and neck cancers but a relatively low risk of lung cancer—which might lead to deviation of the research results [39,40]. Secondly, the data of fruit and vegetable in the cohort were mainly derived from the food frequency questionnaire, which may have selection bias and recall bias that reduce the credibility of the results. This study also did not carry out a detailed classification of vegetable and fruit. Further research should investigate the correlation between specific types of fruit and vegetable and the risk of lung cancer in different smokers. Furthermore, we did not analyze fruit and vegetable separately for men and women with different smoking status, and further studies should explore these differences. Finally, for current smokers, our results found a significant negative relationship between vegetable or fruit consumption and lung cancer risk, but we did not conduct further analysis of their smoking intensity because available data were insufficient. Beyond that, we did not stratify lung cancer subtypes in patients because only a few studies have focused on the correlation between fruit and vegetable consumption and lung cancer subtypes in smokers of different status [8].

## 5. Conclusions

In conclusion, this meta-analysis provides strong evidence that fruit consumption is negatively correlated with lung cancer risk among current smokers and former smokers, while vegetables were significantly negatively correlated with lung cancer risk of current smokers. These findings may have considerable public health significance for the prevention of lung cancer through dietary interventions.

## Figures and Tables

**Table 1:** Characteristics of included studies regarding fruit and vegetable intake and lung cancer risk ^1^.

First Author and Cohort	Publication Year and Region	Age (Gender)	Subjects (Cases)	Follow-Up Period	Exposure Measure	Outcome Measure	Exposure	Covariates Adjusted
Buchner [8], EPIC	2010, Europe	51.2 y M/F	478,535 (1830)	8.7 y	FFQ, dietary questionnaires, food record	Health insurance records, cancer and pathology hospital registries, active follow-up	Fruit and vegetable	Fruits consumption, vegetable consumption, smoking status, duration of smoking, lifetime and baseline intensity of smoking, time since quitting, energy intake, weight, height, alcohol consumption, physical activity, and school level
Feskanich [9], NHS	2000, USA	50.94 y F	77,283 (519)	12 y	FFQ	FFQ, medical records, death certificates	Fruit and vegetable	Age, follow-up cycle, smoking status, years since quitting among past smokers, cigarettes smoked/day among current smokers, age at start of smoking, total energy intake, and availability of diet data after baseline measure
Feskanich [9], HPFS	2000, USA	54.44 y M	47,778 (274)	10 y	FFQ	FFQ, medical records, death certificates	Fruit and vegetable	Age, follow-up cycle, smoking status, years since quitting among past smokers, cigarettes smoked/day among current smokers, age at start of smoking, total energy intake, and Availability of diet data after baseline measure
Fraser [24], AHS	1991, USA,	54.5 y M/W	34,198 (61)	6 y	Dietary questionnaire	Medical record, tumor registries	Fruit and vegetable	Age, sex, and smoking history
Liu [10], JPHC Cohort I	2004, Japan	49.53 y, M	42,224 (177)	10 y	Self-administered questionnaire, FFQ	Histological examination of specimens, biopsy or cytology; clinical findings	Fruit and vegetable	Age, gender, areas, sports, frequency of alcohol intake, BMI, vitamin supplement use, salted fish and meat, pickled vegetables, smoking status, smoking duration, and number of cigarettes per day
Liu [10], JPHC Cohort II	2004, Japan	53.87 y, F	51,114 (251)	7 y	Self-administered questionnaire, FFQ	Histological examination of specimens, biopsy or cytology; clinical findings	Fruit and vegetable	Age, gender, areas, sports, frequency of alcohol intake, BMI, vitamin supplement use, salted fish and meat, pickled vegetables, smoking status, smoking duration, and number of cigarettes per day
Smith-Warner [19]	2003, USA	N M/F	430,281 (3,206)	11 y	FFQ, self-administered questionnaires	Follow-up questionnaires, medical record, cancer registry, mortality registries or death certificates	Fruit and vegetable	Education, BMI, alcohol intake and calories, smoking status, smoking duration for past smokers, smoking duration for current smokers, amount smoked for current smokers
Steinmetz [20], IWHS	1993, USA, Iowa	57 y, F	2952 (138)	4 y	Self-administered questionnaire, FFQ	Health registry, surveillance, epidemiology, and end results program of the National Cancer Institute	Fruit and vegetable	Age, energy intake, and pack-years of smoking
Voorrips [21], NLCS	2000, Netherlands	62 y, M/F	120,852 (1202)	6.3 y	Self-administered questionnaire, FFQ	Regional cancer registries	Fruit and vegetable	Age, sex, family history of lung cancer, highest educational level, current smoker, years of smoking, number of cigarettes per day
Wakai [23]	2015, Japan	54.5 y M/F	190,940 (1742)	12.9 y	Self-administered FFQ, dietary record.	Cancer registries, death certificate	Fruit and vegetable	Age, area, smoking and intake of total energy
Wright [22], NIH-AARP DHS	2008, USA	62 y, M/F	472,081 (6035)	8 y	FFQ	Cancer registries, self-reports and medical records	Fruit and vegetable	Age, energy intake, race, education, BMI, smoking status, smoking dose, time since quitting smoking, alcohol intake, physical activity, and family history of any cancer

^1^: There were nine cohort studies comprising 15,435 lung cancer incident cases among 1,948,238 participants with regard to fruit and vegetable consumption. AHS: Adventist Health Study; EPIC: European Prospective Investigation into Cancer; F: female; FFQ: food-frequency questionnaire; HPFS: Health Professionals Follow-up Study; IWHS: Iowa Women’s Health Study; JPHC: Japan Public Health Center-based Prospective Study; M: male; NHS: Nurses’ Health Study; NLCS: Netherlands Cohort Study; NIH-AARP DHS: National Institutes of Health (NIH)-AARP Diet and Health Study; y, year.

**Table 2:** Quality assessment of each included study according to Newcastle-Ottawa Scale.

Study	Representativeness of the Exposed Cohort	Selection of the Unexposed Cohort	Ascertainment of Exposure	Demonstration that Outcome of Interest at Start of Study	Comparability of Cohorts on the Basis of the Design or Analysis	Outcome Assessment	Follow-Up Long Enough for the Outcomes to Occur	Adequacy of Follow-Up of Cohorts	Total Quality Scores
Buchner		☆		☆	☆	☆	☆		☆☆☆☆☆
Feskanich		☆		☆	☆☆	☆	☆	☆	☆☆☆☆☆☆☆
Fraser		☆		☆	☆☆	☆	☆	☆	☆☆☆☆☆☆☆
Liu		☆		☆	☆☆	☆	☆	☆	☆☆☆☆☆☆☆
Smith-Warner		☆		☆	☆	☆	☆		☆☆☆☆☆
Steinmetz		☆		☆	☆☆	☆			☆☆☆☆☆
Voorrips	☆	☆		☆	☆☆	☆	☆	☆	☆☆☆☆☆☆☆☆
Wakai	☆	☆		☆	☆☆	☆	☆		☆☆☆☆☆☆☆
Wright		☆		☆	☆☆	☆	☆		☆☆☆☆☆☆

The highest rating is 9 stars, and 0–3, 4–6, 7–9 was categorized as low, medium and high-quality, respectively.

## Supplementary Materials

The following are available online at https://www.mdpi.com/2072-6643/11/8/1791/s1, Figure S1: Stratified analysis of current smokers’ fruit consumption by region; Figure S2: Stratified analysis of former smokers’ fruit consumption by region; Figure S3: Stratified analysis of never smokers’ fruit consumption by region; Figure S4: Stratified analysis of current smokers’ vegetable consumption by region; Figure S5: Stratified analysis of former smokers’ vegetable consumption by region; Figure S6: Stratified analysis of never smokers’ vegetable consumption by region; Figure S7: Sensitivity analysis with respect to fruit consumption in current smokers; Figure S8: Sensitivity analysis with respect to fruit consumption in former smokers; Figure S9: Sensitivity analysis with respect to fruit consumption in never smokers; Figure S10: Sensitivity analysis with respect to vegetable consumption in current smokers; Figure S11: Sensitivity analysis with respect to vegetable consumption in former smokers; Figure S12: Sensitivity analysis with respect to vegetable consumption in never smokers.
