# Supplementary material for: The Associations of Fruit and Vegetable Intake with Lung Cancer Risk in Participants with Different Smoking Status: A Meta-Analysis of Prospective Cohort Studies
Source: Nutrients. 2019 Aug 2;11(8):1791. doi: 10.3390/nu11081791 (PMC6723574; doi:10.3390/nu11081791)
Supplement: Supplementary file 1 [file nutrients-11-01791-s001.pdf]

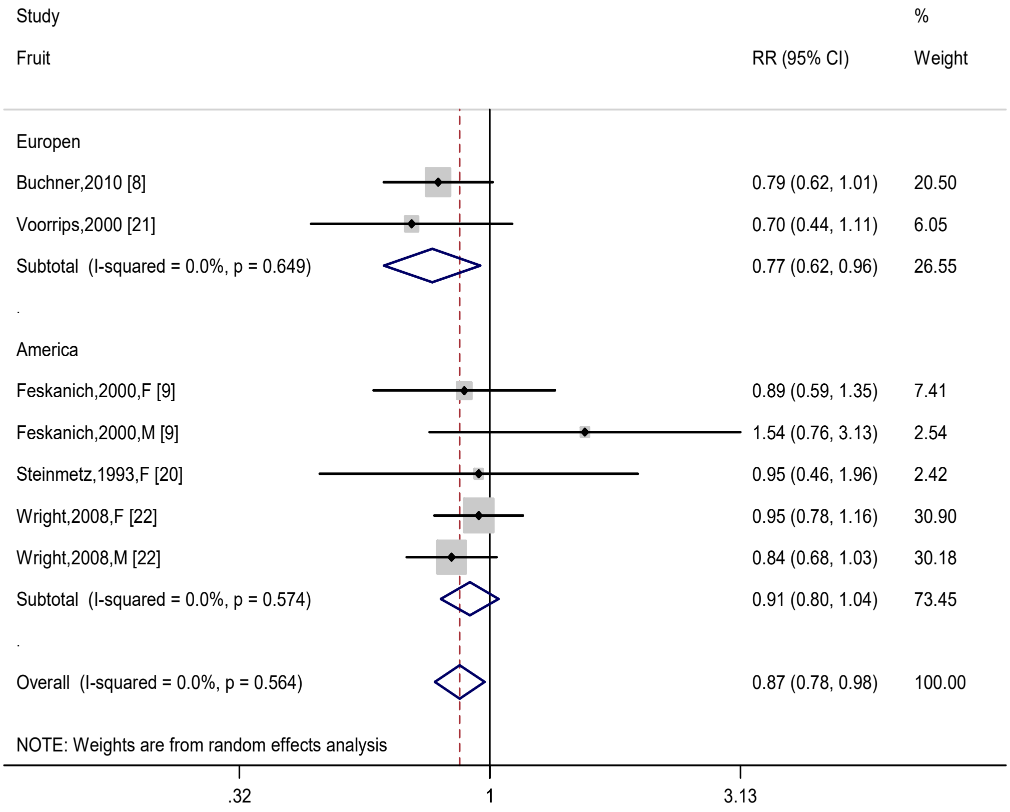

Figure S1. Stratified analysis of current smokers' fruit consumption by region.

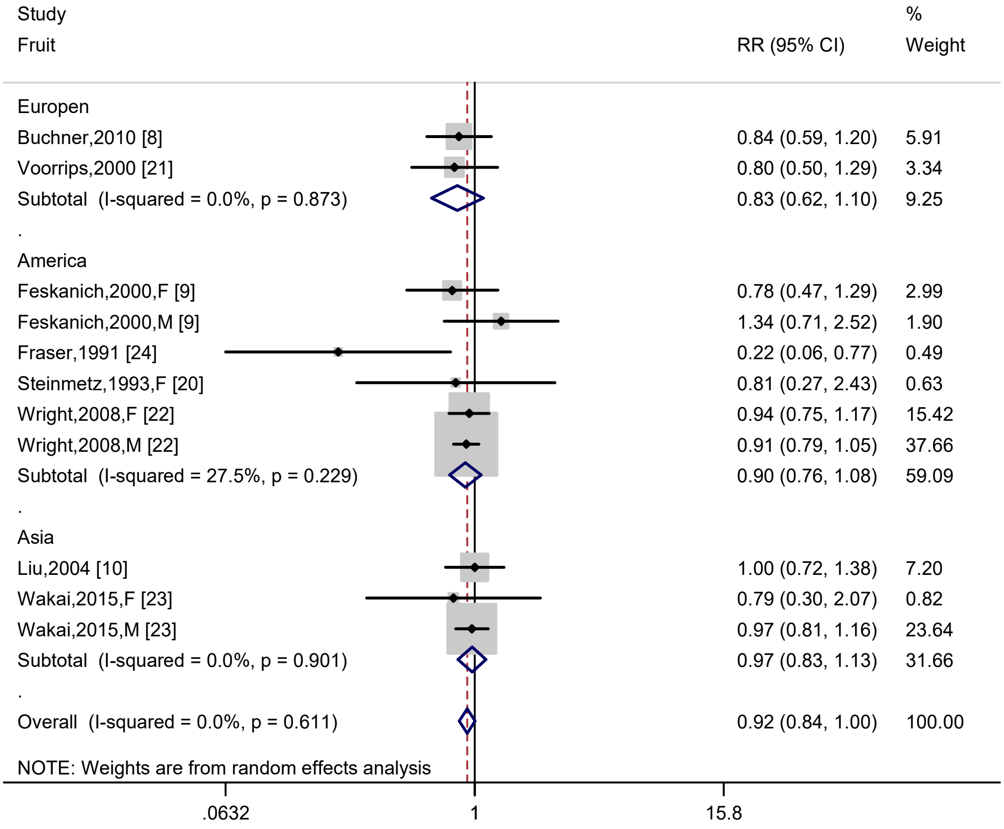

Figure S2. Stratified analysis of former smokers' fruit consumption by region.

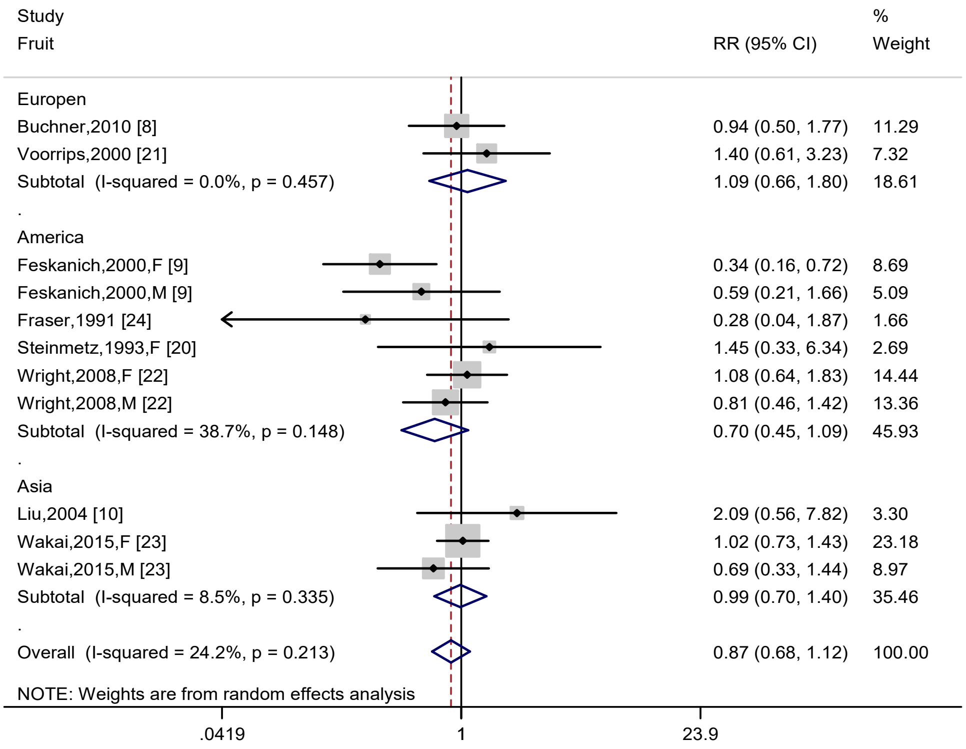

Figure S3. Stratified analysis of never smokers' fruit consumption by region.

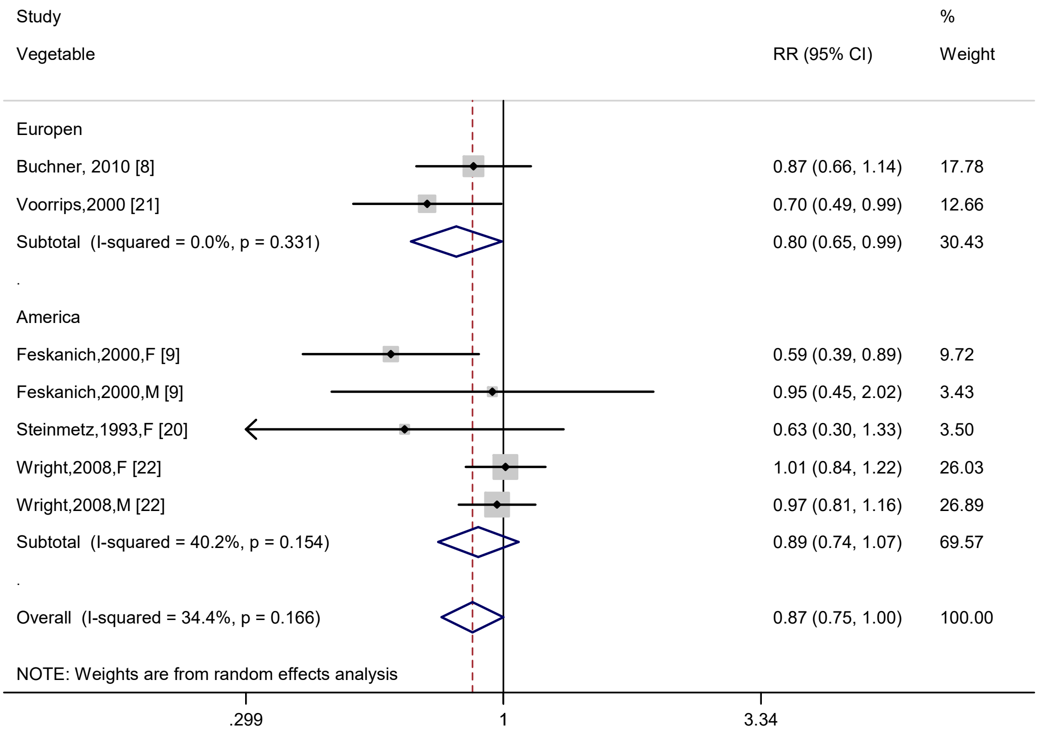

Figure S4. Stratified analysis of current smokers' vegetable consumption by region.

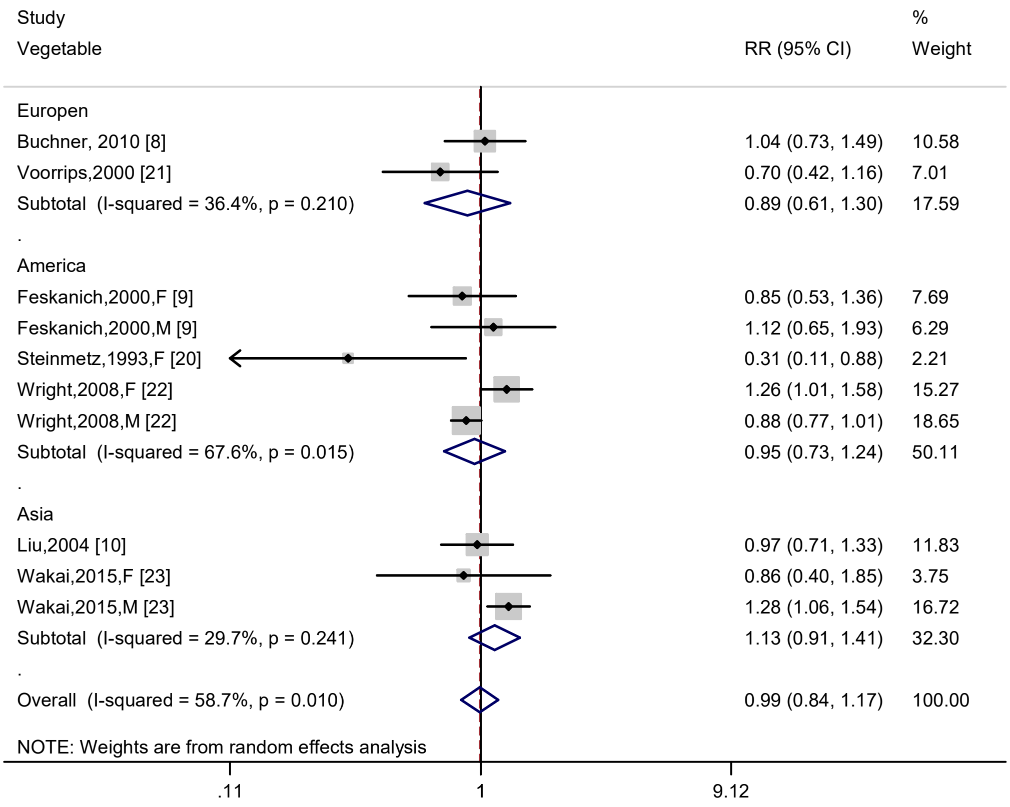

Figure S5. Stratified analysis of former smokers' vegetable consumption by region.

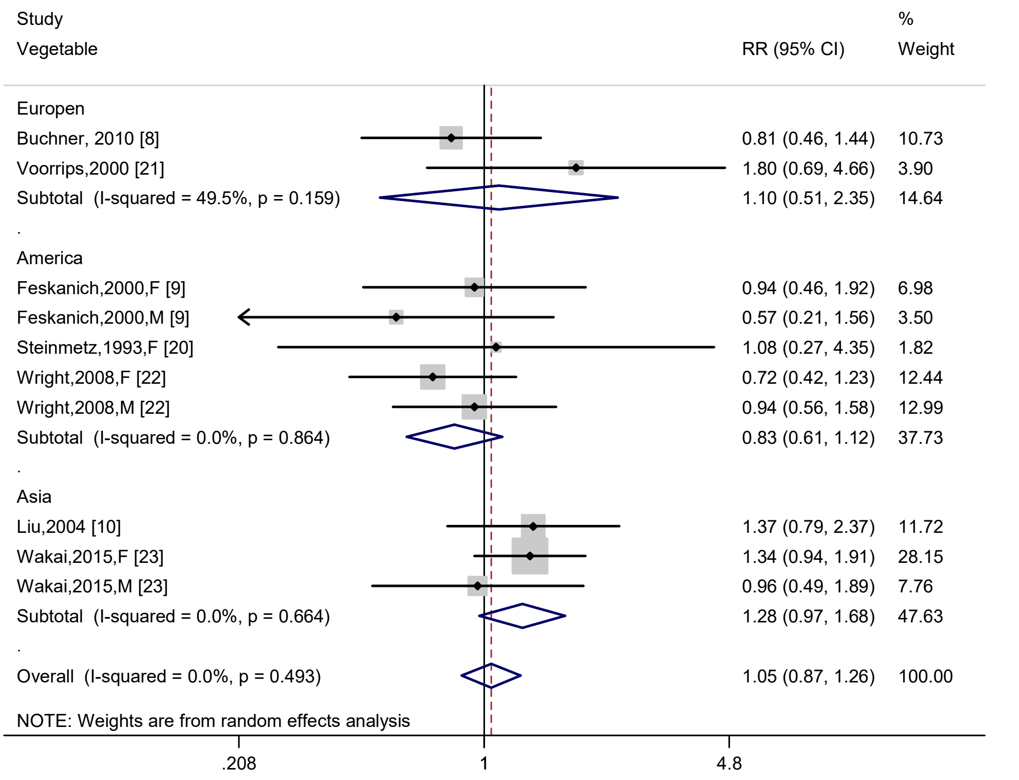

Figure S6. Stratified analysis of never smokers' vegetable consumption by region.

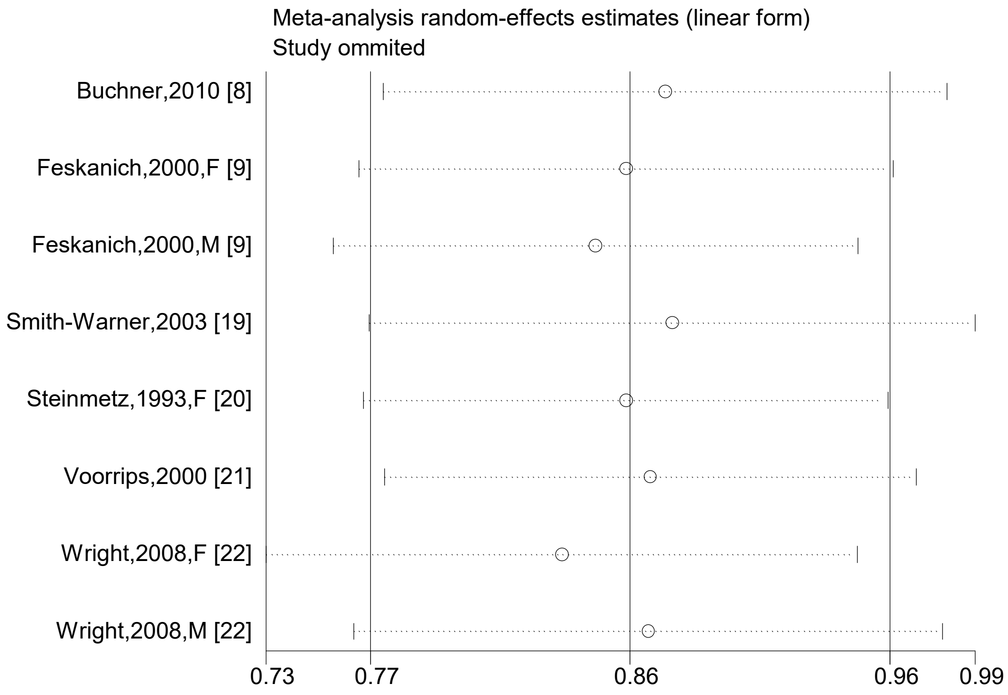

Figure S7. Sensitivity analysis with respect to fruit consumption in current smokers.

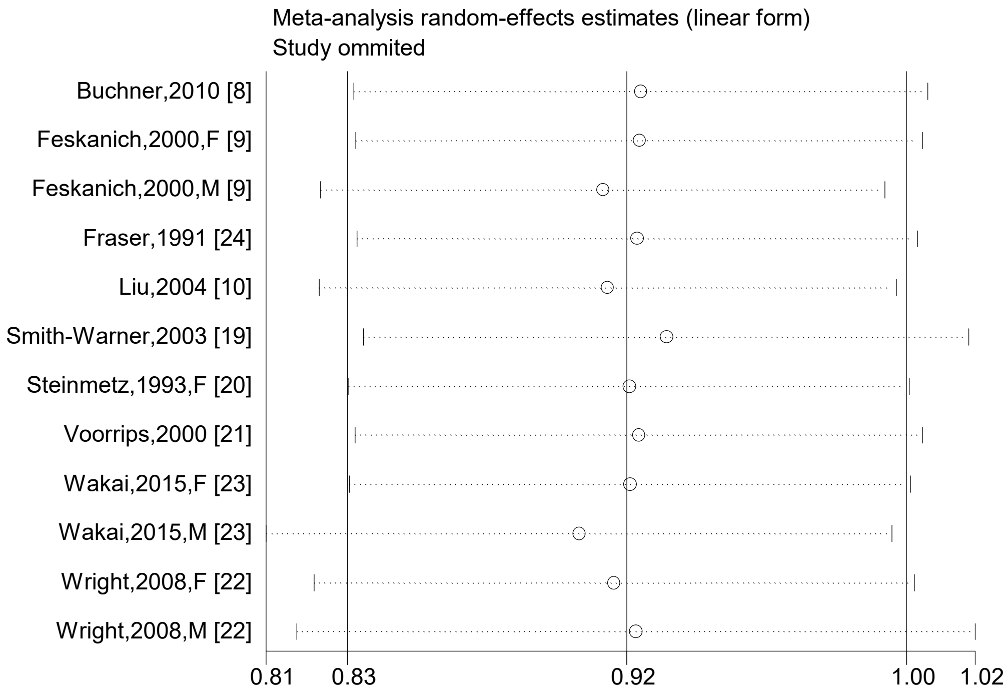

Figure S8. Sensitivity analysis with respect to fruit consumption in former smokers.

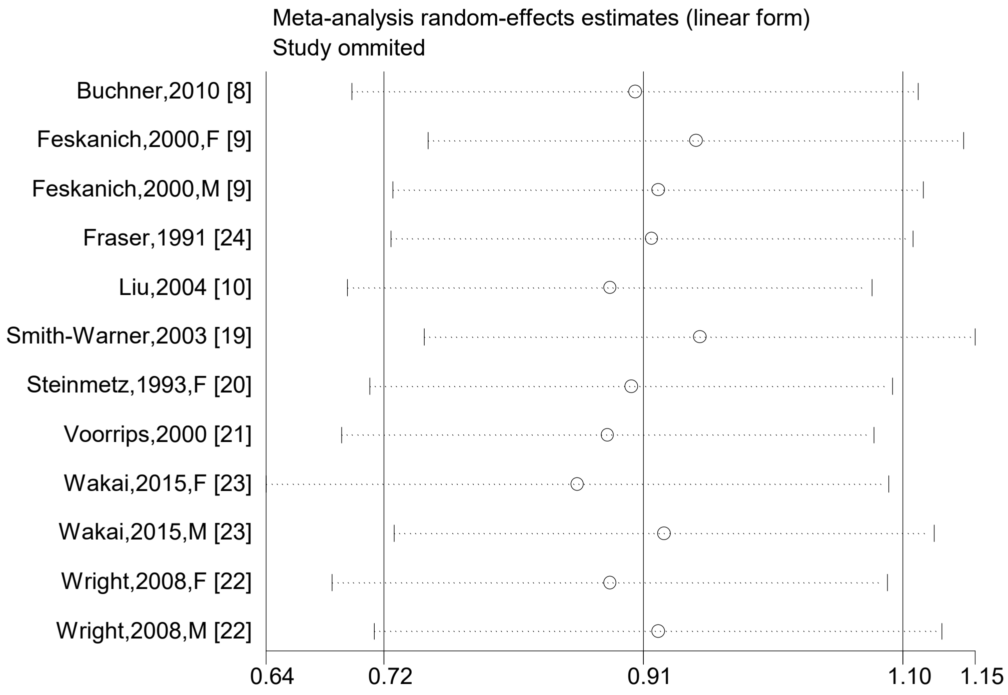

Figure S9. Sensitivity analysis with respect to fruit consumption in never smokers.

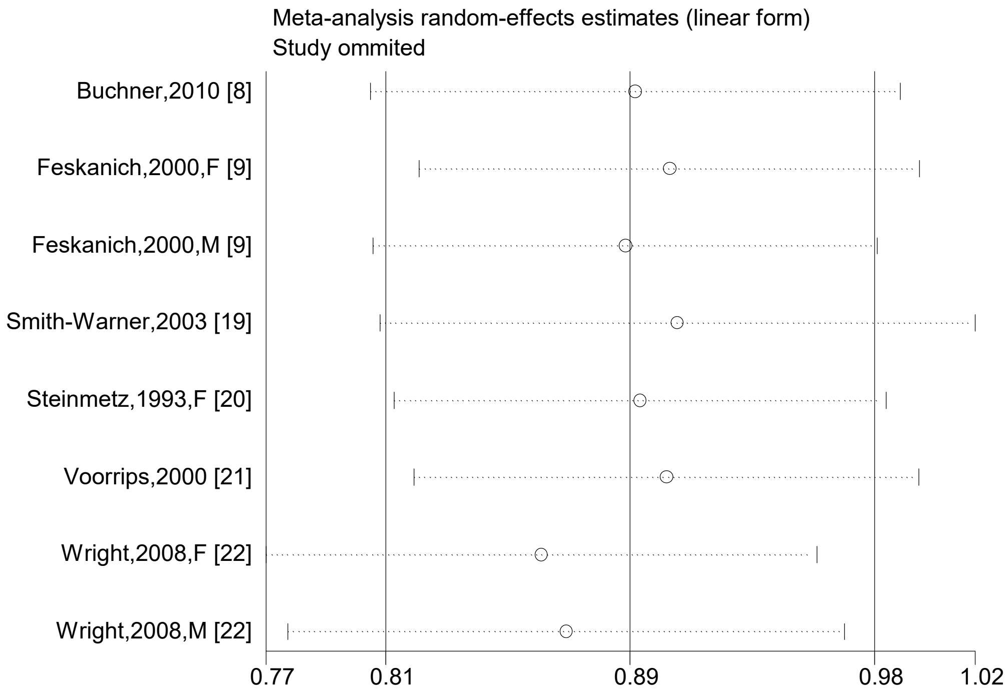

Figure S10. Sensitivity analysis with respect to vegetable consumption in current smokers.

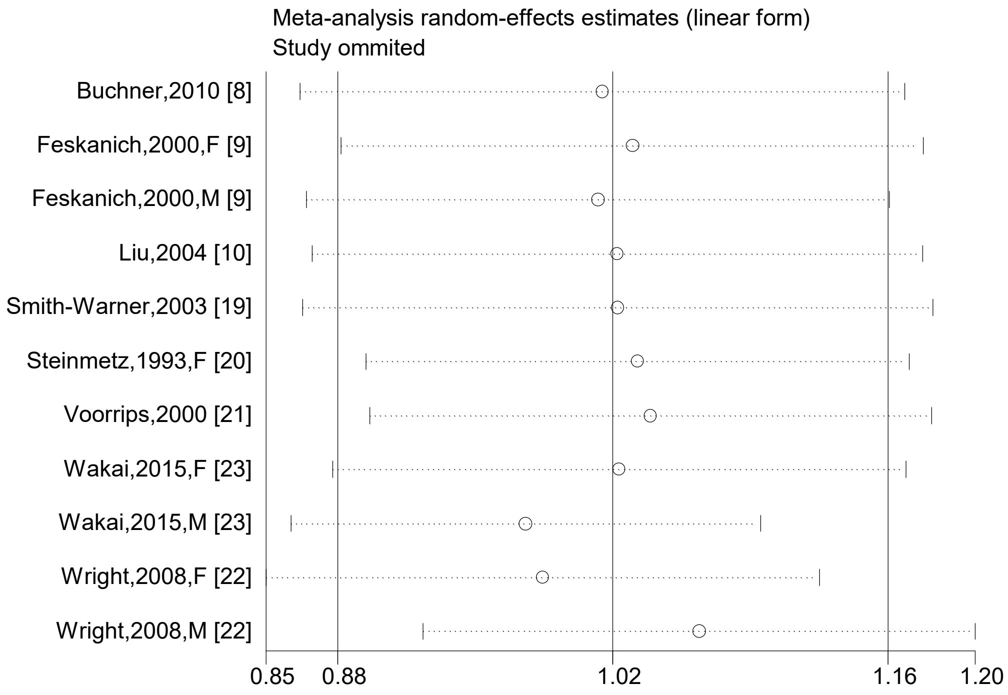

Figure S11. Sensitivity analysis with respect to vegetable consumption in former smokers.

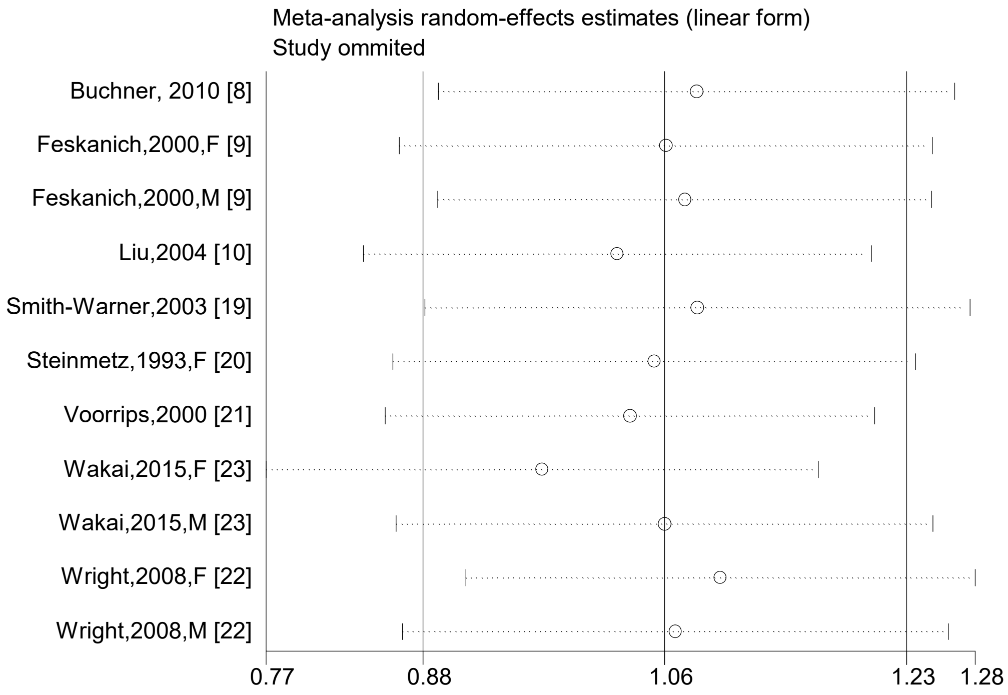

Figure S12. Sensitivity analysis with respect to vegetable consumption in never smokers.
